# Application of improved transformer based on weakly supervised in crowd localization and crowd counting

**DOI:** 10.1038/s41598-022-27299-0

**Published:** 2023-01-20

**Authors:** Hui Gao, Wenjun Zhao, Dexian Zhang, Miaolei Deng

**Affiliations:** 1 College of Mechanical and Electrical Engineering, Henan University of Technology, Zhengzhou, 450001 China; 2 Henan International Joint Laboratory of Grain Information Processing, Zhengzhou, 450001 China; 3 College of Information Science and Engineering, Henan University of Technology, Zhengzhou, 450001 China

**Keywords:** Electrical and electronic engineering, Mechanical engineering

## Abstract

To the problem of the complex pre-processing and post-processing to obtain head-position existing in the current crowd localization method using pseudo boundary box and pre-designed positioning map, this work proposes an end-to-end crowd localization framework named WSITrans, which reformulates the weakly-supervised crowd localization problem based on Transformer and implements crowd counting. Specifically, we first perform global maximum pooling (GMP) after each stage of pure Transformer, which can extract and retain more detail of heads. In addition, we design a binarization module that binarizes the output features of the decoder and fuses the confidence score to obtain more accurate confidence score. Finally, extensive experiments demonstrate that the proposed method achieves significant improvement on three challenging benchmarks. It is worth mentioning that the WSITrans improves F1-measure by 4.0%.

## Introduction

Crowd localization and crowd counting are important subtasks of crowd analysis, which play a crucial role in crowd monitoring, traffic management, and commerce. Most of the algorithms get crowd counting by regressing the predicted density map, which has achieved significant progress. However, crowd localization is more conducive to public safety management in crowd detection and crowd tracking. Therefore, crowd localization has become a new branch of computer vision and attracted a lot of attention from researchers.

For a long time, crowd counting has achieved rapid development, and researchers have put forward many effective crowd counting methods. Detection-based methods^1–3^ use box-level annotated supervised detectors for predicting the head center position in sparse scenarios. In dense scenes, regression-based methods^4,5^ output image-level numbers by summing the predicted density maps. With the development of deep learning, Transformer has been rapidly spread in the field of computer vision, and the ViT-based crowd counting approaches have achieved remarkable results, such as TransCrowd^6^, BCCT^7^, CCTrans^8^, Twin SVT^9^, and SMS^10^. However, most existing methods only focus on the crowd counting task but do not implement the crowd localization task in crowd analysis.

To solve the above problem, we propose an improved Transformer method based on weak supervision, which only focuses on the center position of the head, not only does not need to annotate the frame of each head but also does not need these annotations in the evaluation, so as to improve the performance of crowd analysis such as crowd positioning and crowd counting. The main contributions of our work are as follows.

1. We propose an end-to-end crowd localization framework named WSITrans, which reformulates the weakly-supervised crowd localization problem based on Transformer and implements crowd counting.
2. To obtain more abundant head details, we improve the backbone network that performs a global maximum pooling operation after each stage of the extraction feature.
3. We design a binarization module, which binarizes the output features of the decoder with the fusion of confidence score to obtain a more accurate confidence score. Moreover, extensive experiments illustrate that our approach has achieved a consistent improvement on three challenging benchmarks.

## Related work

### Vision transformer (ViT)

With the rapid development of deep learning, Transformer has spread rapidly in computer vision. To be specific, Carion et al.^11^ proposed an end-to-end trainable detector transformer (DETR) without NMS. The transformer decoder was used to model the target detection in the end-to-end pipeline, and only one single-stage feature map was used to successfully eliminate the need for post-processing and achieve competitive performance. However, DETR mainly relies on L_1_ distance with class confidence, that is, assigning each independent match to each ground truth (GT) without context may lead to errors. Different from target detection, crowd images only contain one category of a human head, while dense heads have a similar texture, so the prediction reliability is high, which greatly reduces the positioning effect of the algorithm. Motivated by DETR, Meng et al.^12^ proposed a conditional cross-attention mechanism for fast DETR training, which accelerated the convergence of DETR. In crowd analysis, Liang et al.^6^ proposed TransCrowd, which expressed the weakly supervised crowd counting problem from the perspective of sequence counting based on ViT. TransCrowd can effectively extract semantic crowd information by using a self-attention mechanism of ViT. In addition, this is the first time that researchers have used ViT to conduct crowd counting research, and achieved significant results. Sun et al.^7^ showed the function of the Transformer in the point monitoring crowd counting setting. However, they all focused on the crowd counting task, not the crowd positioning task.

### Weakly-supervised

Only a few methods focus on counting with a lack of labeled data. There is no point-level annotation with data, or the number of point-level annotations is limited. Lei et al.^13^ learned the model from a small number of point-level annotations (fully supervised) and a large number of count level annotations (weakly supervised). Borstel et al.^14^ proposed a weak supervised solution based on the Gaussian process for crowd density estimation. Similarly, Yang et al.^15^ proposed a soft label-sorting network, which can directly return the number of people without any localization monitoring. Meanwhile, most crowd localization methods are based on density maps, such as distance label map^16^, focal inverse distance transform map (FIDTM)^17^ and independent instance map (IIM)^18^. However, these density map-based methods require complex and non-differentiable post-processing to extract the head position, such as "find maximum value". In addition, density map-based methods rely on high-resolution representation to generate a clear map to better find the local maximum, which means that multiscale feature map is needed.

## Methodology

**Figure 1:**
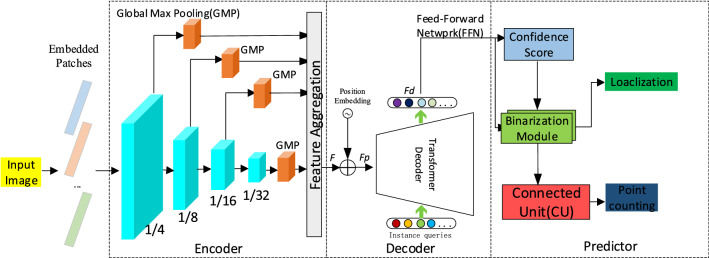
The Architecture of the Proposed WSITrans. The method can directly predict all instances without additional preprocessing and post-processing, including encoder, decoder, and predictor.

To solve the issues of concern, we firstly apply the pure Transformer model to crowd localization, and propose an improved transformer framework, is called WSITrans which based on weakly supervised, as shown in Fig. 1. This method can directly predict all instances without additional preprocessing and post-processing, it consists of three subnetworks, encoder network, decoder network, and predictor. Specifically, firstly, the multiscale features are extracted from the input image using the pre-trained transformer backbone network. After the GMP operation, the combined feature F is obtained through the aggregation module. Secondly, the feature F_p_ after position embedding of the combined features is input into the decoder, a set of trainable embedding is used as a query in each decoder layer, and visual features of the last layer of the encoder are taken as keys and values, and decoding feature F_d_ is output to predict the confidence score. Finally, the scores of F_d_ and confidence score are sent to the threshold learner of the binarization module, and the confidence map is accurately binarized, so we can get the center position of the head.

### Transformer backbone network

The WSITrans adopts the pyramid vision transformer as the backbone network for feature extraction. Here, we refer to the " PVTv2 B5"^19^, as shown in Table 1. It includes four stages, and each stage generates feature maps of different scales to perform a GMP operation. The architecture of each phase consists of an overlapping patch embedding layer and *L*_*i*_ number of transformer encoder layer, which is *L*_*i*_ encoder layer of the i-th stage. PVTv2 uses overlapping patch embedding to label images. When the patch is generated, the overlapping area of adjacent windows is half of its area. Overlapping patch embedding is realized by applying zero-padding convolution and appropriate step size. Specifically, for the size of W × H × C, The input of C, the kernel size of the convolution layer is 2S-1, the zero padding is S-1, the step size is S, and the number of cores C is used to generate an output size of $\frac{H}{S}\times \frac{W}{S}\times C$. In the first stage, the convolution step of patch generation is S = 4, and the rest is S = 2. Therefore, we obtain a set of feature maps from the i-th stage, which is 2^(*i*+1)^ smaller than the size of the input image.

**Table 1:** The parameters of encoder of WSITrans network.

Step	Output size	Layer name	B5
Stage 1	$\frac{H}{4}\times \frac{W}{4}$	Overlapping patch embedding	S_1_ = 4
			C_1_ = 64
		Transformer encoder	R_1_ = 8
			N_1_ = 1
			E_1_ = 4
			L_1_ = 3
		GMP	
Stage 2	$\frac{H}{8}\times \frac{W}{8}$	Overlapping patch embedding	S_2_ = 2
			C_2_ = 128
		Transformer encoder	R_2_ = 4
			N_2_ = 2
			E_2_ = 4
			L_2_ = 6
		GMP	
Stage 3	$\frac{H}{16}\times \frac{W}{16}$	Overlapping patch embedding	S_3_ = 2
			C_3_ = 320
		Transformer encoder	R_3_ = 2
			N_3_ = 5
			E_3_ = 4
			L_3_ = 40
		GMP	
Stage 4	$\frac{H}{32}\times \frac{W}{32}$	Overlapping patch embedding	S_4_ = 2
			C_4_ = 512
		Transformer encoder	R_4_ = 1
			N_4_ = 8
			E_4_ = 4
			L_4_ = 3
		GMP	

It refers to the “PVTv2 B5”, and we perform global maximum pooling (GMP) at the end of stage i.

### Encoder

The encoder uses a 1-D sequence as input, the feature F_p_ extracted from the transformer backbone network can be directly sent to the transformer encoder layer to generate the encoding feature F_e_. Here, the encoder consists of four standard Transformer layers, each of which includes a self-attention (*SA*) layer and a feed-forward (*FF*) layer. *SA* consists of three inputs, including query (*Q*), key (*K*), and value (*V*), which are defined as follows.

1 $$SA\left(Q, K, V\right)=softmax \left(\frac{Q{K}^{T}}{\sqrt{c}}\right)V$$

where, *Q, K*, and *V* are obtained from the same input *Z* (e.g., *Q* = *ZWQ*). In particular, we employ a multi-self-attention (*MSA*) to model complex feature relationships, which is an extension of several independent *SA* modules: *MSA* = [*SA*_1_; *SA*_2_; ···; *SA*_m_]*W*, where *W* is the projection matrix and *m* is the number of attention heads set to 8.

#### Standard transformer

**Figure 2:**
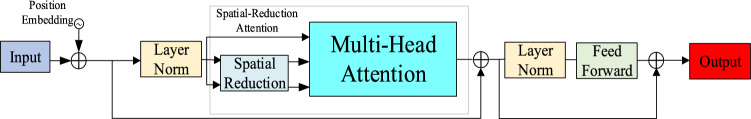
The architecture of standard transformer.

The standard Transformer stage consists of spatial-reduction attention (*SRA*), feedforward (*FF*) blocks, and layer norm (*LN*), as shown in Fig. 2. At the beginning of stage i, the input is evenly divided into overlapping patches of equal size, and each patch is flattened and projected into the C_i_ dimension embedding. These dimensions are embedded in stages 512, 320, and 64, respectively. Each encoder consists of an *SRA* and a *FF*. The position embedding is completed before the transformer encoder. In WSITrans, the input image size is 384 × 384 × 3 pixels, and the patch size of the first stage is 7 × 7 × 3 and 3 × 3 × C_i_, where Ci is the embedded dimension of the i-th stage. As mentioned earlier, C_2_ = 64, C_3_ = 128, and C_4_ = 320. Therefore, the sizes of the output features are 96 × 96 × 64, 48 × 48 × 128, 24 × 24 × 320, and 12 × 12 × 512.

By experimental comparison, we found that the localization effect of GMP is better than that of global average pooling (GAP). Therefore, we obtain the feature map from each stage, perform a GMP operation to obtain 1-D sequences of dimensions 64, 128, 320, and 512, and project each of these sequences into a 1-D sequence with a length of 6912.

### Decoder

The transformer decoder consists of several decoder layers; each layer consists of three sub-layers: (1) Self-attention (*SA*) layer. (2) Cross attention (*CA*) layer. (3) Feedforward (*FF*) layer. *SA* and *FF* are the same as encoders. The *CA* module takes two different embeddings as input instead of the same input in *SA*. Let's express the two embeddings as *X* and *Y*, *CA* can be written as follows.

2 $$CA = SA\left( {q \, = \, XWQ, \, k \, = \, YWK, \, v \, = \, YWV} \right)$$

In this paper, each decoder uses a set of trainable embeddings as queries, and the visual features of the last encoder layer are used as keys and values. The decoder outputs the decoded feature, which is adopted to predict the point coordinate and the confidence score of the human head, so as to obtain the number of people and crowd localization in the scenario.

### Predictor

#### Binarization module

**Figure 3:**
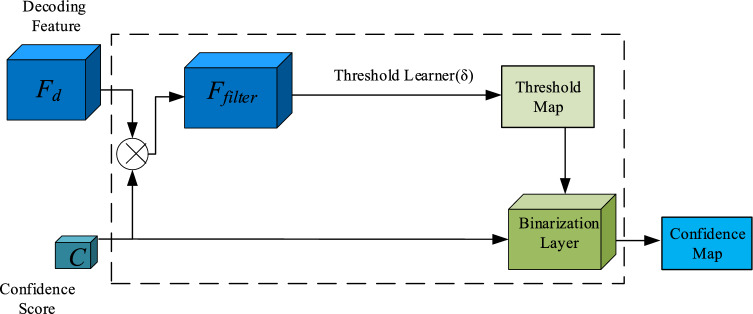
The Flowchart of Binarization Module. The attention filter is a dot product operation between the decoding feature F_d_ and predicted confidence map C.

Many mainstream methods use thermal maps to locate targets, usually setting thresholds to filter localization information from the predicted heat maps. Most heuristic crowd localization methods^2,3,17,20^ use a single threshold to extract the head points on the dataset. This is not the best choice because the confidence response between low confidence and high confidence is different. To alleviate this problem, IIM proposed learning a pixel-level threshold map to segment the confidence map, which can effectively improve the capture of lower response heads and eliminate the overlap in adjacent heads. However, there are two problems: (1) threshold learners may induce not a number (NaN) phenomenon during training. (2) The predicted threshold map is relatively rough. Therefore, we consider redesigning the binarization module to solve these two problems. As shown in Fig. 3, the confidence score is fed into the threshold learner for decoding the pixel-level threshold map^21^.

Here, we perform pixel-level attention filter operation instead of directly passing feature map F_d_. The attention filter can be represented as follows.

3 $${F}_{filter}={F}_{d } \otimes D \left(C, \frac{1}{4}\right)$$

where, *D*(*x, y*) is a downsampling function, indicating to change the size of *x* to *y* × of the input image.

The core components of the binarization module are the threshold learner and binarization layer. The former learns pixel-level threshold map T from the filter, while the latter confidence map C into binarization map B. The threshold learner is composed of five convolution layers: the first three layers are composed of 3 layers, and each layer has a batch normalization and ReLU activation function. The kernel size of the last two layers is 3 × 3 and 1 × 1, then batch normalization, ReLU, and average pool layer. Add window size to 9 × 9 to smooth the threshold graph. Finally, a custom activation function is introduced to solve the NaN phenomenon^18^.

4 $$f\left(x\right)= P({T}_{i,j}\le x)=\left\{ \begin{array}{ll}0.25 & \quad x<0.25\\ 0.90 & \quad x>0.90\\ x & \quad otherwise\end{array}\right.$$

Equation (4), ${T}_{i,j}$ is limited to [0.25, 0.90]. Compared with the compressed Sigmoid, it does not force the last layer to output meaningless values such as ± ∞, so it increases the stability of numerical calculation. To ensure that the threshold is properly optimized in the training process, Eq. (5) provides the derivation rules of Eq. (4).

5 $$\frac{\partial f}{\partial x}=\left\{\begin{array}{ll}{e}^{x-0.25} & \quad x<0.25\\ 0 & \quad x>0.90\\ 1 & \quad otherwise\end{array}\right.$$

The threshold learner is defined as δ, parameter is ${\theta }_{t}$. The output threshold map is shown in Eq. (6).

6 $${\rm T}=\updelta ({F}_{filter}; {\theta }_{t})$$

Now, we obtain the function by forwarding the confidence map *C* and the threshold map *T* to the differentiable binarization layer, (*C*, *T*). The formula is as follows.

7 $${B}_{i, j}= \o \left(C,T\right)= \left\{\begin{array}{ll}1, & \quad {C}_{i, j} \ge {T}_{i,j}\\ 0, & \quad otherwise\end{array}\right.$$

### Connecting unit

After obtaining the binarization map *B*, localization and counting are equivalent to detecting connected unit from *B*, where each blob corresponds to an instance. We set $R= \left\{\left({x}_{i}, {y}_{i},{w}_{i},{h}_{i} \right)|(i = 1\dots N)\right\}$ as the connected unit that contains a set of binarization maps, and the blob $\left({x}_{i}, {y}_{i}\right)$ is the center point of the object, ${w}_{i}$ and ${h}_{i}$ are the width and height of the blob. Then, the points set $P= \left\{\left({x}_{i}, {y}_{i}\right)|(i = 1\dots N)\right\}$ is the position result, and the number of points is regarded as the counting result.

## Experiments

### Datasets

We evaluated our approach on three challenging datasets that are publicly available for crowd counting and can be downloaded from the Internet. The three datasets are detailed as follows.

*ShanghaiTech*^22^ is one of the largest large-scale population statistical datasets in previous years, consisting of 1198 images and 330,165 annotations. According to the density distribution, the dataset is divided into PartA and PartB. The training and test images consist of 182 images. PartB includes 400 training images and 316 test images. PartA is a random selection of images from the Internet, and PartB is a picture taken from a busy street in a metropolis in Shanghai. The density in PartA is much higher than that in PartB. The scale variation and perspective distortion presented by this dataset provide new challenges and opportunities for the design of many CNN-based networks.

*UCF_QNRF*^4^ is a dense dataset containing 1535 images (1201 for training and 334 for testing) and 1,251,642 annotations. The average number of pedestrians per image is 815, and the maximum number is 12,865. The images in this dataset have a wider range of scenes and contain the most diverse set of viewpoints, density, and illumination changes.

*NWPU-Crowd*^23^ is a large-scale dataset collected from various scenes, including 5109 images and 2,133,238 annotated instances. These images are randomly divided into a training set, validation, and test set, which contain 3,109,500 and 1500 images respectively. In addition to the amount of data, there are other advantages over the previous data sets in the real world. It includes negative samples, fair evaluation, higher resolution, and larger appearance changes. This dataset provides point-level and frame-level annotations.

### Training details

#### Implementation

For the above data sets, the original size images were randomly flipped horizontally, scaled (0.8–1.2 times,) and cropped (768 × 1024) to increase training data. The batch size is 8, the binarization module learning rate is set to 1e−5, and the learning rate of other learnable modules is initialized to 1e−6. During the training period, we optimize the decay rate of Adam^27^. We choose the best model in the verification set to test and evaluate our model. We divide 10% of the training dataset into a verification set. In the test phase, we select the best-performing model on the verification set to evaluate the performance on the test set. We perform end-to-end prediction without multiscale prediction fusion and parameter search.

#### Loss function

After obtaining the one-to-one matching result, we need to calculate the backpropagation loss. Since the number of people in different images varies greatly, and *L*_1_ loss^22^ is very sensitive to outliers, we use smooth Loss ${L}_{s}$, not ${L}_{1}$ loss. Smooth Loss ${L}_{s}$ can be calculated by using (8).

8 $${L}_{s}=\left\{\begin{array}{ll}\frac{{({Pre}_{i}-{Gt}_{i})}^{2}}{2\beta }, & \quad |{Pre}_{i}-{Gt}_{i}|\le \beta \\ \left|{Pre}_{i}-{Gt}_{i}\right|-0.5\times \beta , & \quad otherwise\end{array}\right.$$

In formula (8), when $\left|{Pre}_{i}-{Gt}_{i}\right|>\beta$, ${L}_{s}$ is *L*_1_ loss. When $|{Pre}_{i}-{Gt}_{i}|\le \beta$, ${L}_{s}$ is *L*_1_ loss. $\beta$ is a super parameter, ${Pre}_{i}$ and ${Gt}_{i}$ indicate the predicted value of people and the GT in a given image, separately.

### Evaluation criteria

This research focuses on crowd localization, and counting is an incidental task. The evaluation criteria consist of localization criteria and counting criteria.

#### Localization criteria

In this work, we use precision (Pre), recall (Rec), and F1-measure (F1) as evaluation indicators of crowd localization. The specific calculations are as follows.

9 $$\mathrm{Pre}= \frac{TP}{TP+FP}$$

10 $$\mathrm{Rec}= \frac{TP}{TP+FN}$$

11 $$F1= \frac{2*Pre*Rec}{Pre+Rec}$$

Among them, true positive (*TP*) represents the number of predicted positive samples and actual positive samples, and predicts the correct number; false positive (*FP*) is the number of prediction errors when the prediction is positive but the actual is negative; false negative (*FN*) refers to the number of prediction errors for negative samples but positive samples. Prediction points and GT follow a one-to-one match. If the distance of the matching alignment is less than the distance threshold σ, the corresponding prediction point is regarded as the position of the center point of the head. For ShanghaiTech, we use two fixed thresholds to include σ = 4 and σ = 8. For UCF_QNRF, we use various threshold ranges in [1, 2, 3, 4, …, 100], similar to CL^4^. For NWPU group datasets that provide box-level annotation, σ set to $\sqrt{{w}^{2}+{h}^{2}}/2$, where *w* and *h* indicate the width and height, respectively.

#### Counting criteria

Mean absolute error (MAE) and root mean square error (RMSE) is used as the evaluation criteria for counting, it can be defined as follows.

12 $$MAE=\frac{1}{N}\sum_{i=1}^{N}|{Pre}_{i}-{Gt}_{i}|$$

13 $$RMSE=\sqrt{\frac{1}{N}\sum_{i=1}^{N}{|{Pre}_{i}-{Gt}_{i}|}^{2}}$$

where, *N* is the sum of images, ${Pre}_{i}$, and ${Gt}_{i}$ indicate the predicted value and the GT in the i-th image, respectively.

#### Ablation

We examine the impact of varying the size of the Transformer, including the number of encoder/decoder layers and trainable instance queries. As shown in Table 2, the WSITrans achieves the best performance when the number of layers and queries are set to 6 and 500, separately. When the number of queries is 300, the accuracy of the proposed WSITrans reduces to 74.5%. When the number of queries is changed to 700, the accuracy of the proposed method decreases to 74.3%. Therefore, too many or too few queries will affect the performance of the proposed algorithm.

**Table 2:** Effect of transformer size on ShanghaiTech PartA dataset.

Layers	N (queries number)	Params	Localization (σ = 8)		
			Pre (%)	Rec (%)	F1 (%)
3	500	33.1	70.1	71.1	71.2
6	500	43.2	74.9	73.6	74.3
12	500	62.2	71.2	71.6	72.1
6	300	43.1	74.5	73.2	74.1
6	500	43.2	74.9	73.6	74.3
6	700	43.3	74.3	73.2	73.9

## Results and discussion

### Results of crowd localization

We first used some of the most advanced methods to evaluate localization performance. For NWPU-Crowd, as shown in Table 3, for a large dataset, the F1 measurement value of WSITrans proposed in this paper is better than AutoScale^24^, at least 4.0%. It is worth noting that this dataset provides precise box-level annotations. Although this method is merely based on point annotation, it is a weaker marking mechanism. However, it can still achieve competitive performance on the NWPU-Crowd test set. For the dense data set UCF_QNRF (see Table 4), this method achieves the best recall and F1 measure. For ShanghaiTech PartA (see Table 5), a sparse dataset, our WSITrans improves the most advanced method TopCount by 2%. F1 for strict settings (σ = 4), and less stringent settings (σ = 8) are ill excellent. The experimental results show that the proposed approach can deal with large-scale, dense, and sparse scenes.

**Table 3:** Localization performance on NWPU-Crowd dataset.

Method	Validation set			Test set		
	Pre (%)	Rec (%)	F1 (%)	Pre (%)	Rec (%)	F1 (%)
Faster RCNN^25^^a^	96.4	3.8	7.3	95.8	3.5	6.7
RAZ^2,6^	69.2	56.9	62.5	66.6	54.3	59.8
AutoScale^24^	70.1	63.8	66.8	67.3	57.4	62.0
WSITrans (ours)	70.9	68.3	71.8	70.1	62.2	66.0

^a^Means the methods rely on box-level instead of point-level annotations.

**Table 4:** Localization performance on the UCF-QNRF dataset.

Method	Pre (%)	Rec (%)	F1 (%)
TopCount^20^^a^	81.77	78.96	80.34
CL^4^	75.80	59.75	66.82
LSC-CNN^2^	75.84	74.69	75.26
AutoScale^24^	81.31	75.75	78.43
WSITrans (ours)	82.02	78.60	80.77

^a^Means the methods rely on box-level instead of point-level annotations.

**Table 5:** Localization performance on the ShanghaiTech PartA dataset.

Method	σ = 4			σ = 8		
	Pre (%)	Rec (%)	F1 (%)	Pre (%)	Rec (%)	F1 (%)
LSC-CNN^2^	33.4	31.9	32.6	63.9	61.0	62.4
Method^27^	34.9	20.7	25.9	67.7	44.8	53.9
TopCount^20^^a^	41.7	40.6	41.1	74.6	72.7	73.6
LCFCN^28^	43.3	26.0	32.5	75.1	45.1	56.3
WSITrans (ours)	45.7	41.9	42.2	74.7	73.1	72.2

^a^Means the methods rely on box-level instead of point-level annotations.

### Results of crowd counting

In this paper, we get the number of crcrowdshile implementing the crowd localization task. In this section, we compare the crowd counting performance of localization-based methods, as shown in Table 6. Although our approach only inputs 1/32 feature maps of the original image, it can achieve significant performance in all experiments. Specifically, our method implements the first RMSE and the second MAE on the NWPU-Crowd testset. Compared with the serval crowd counting method that can provide localization information, our method achieves the best performance in MAE and RMSE of ShanghaiTech PartA and PartB datasets. On the UCF_QNRF dataset, our approach achieves the best RMSE and reports comparable MAE.

**Table 6:** Crowd counting performance on the ShanghaiTech, UCF_QNRF, and NWPU-Crowd dataset.

Method	ShanghaiTech				UCF_QNRF		NWPU-Crowd			
	PartA		PartB				Validation set		Test set	
	MAE	RMSE	MAE	RMSE	MAE	RMSE	MAE	RMSE	MAE	RMSE
RAZ^2,6^	71.6	120.1	9.9	15.6	118.0	198	128.7	665.4	151.4	634.6
TopCount^20^	61.2	104.6	7.8	13.7	89.0	159.0	–	–	107.8	438.5
AutoScale^24^	65.8	112.1	8.6	13.9	104.4	174.2	97.3	571.2	123.9	515.5
GL^29^	61.3	95.4	7.3	11.7	84.3	147.5	–	–	79.3	346.1
CLTR^30^	56.9	95.2	6.5	10.6	87.3	142.4	51.7	137.0	84.4	344.4
WSITrans (ours)	54.1	97.3	7.1	9.9	86.5	140.3	50.6	153.8	80.1	331.0

### Visualization

**Figure 4:**
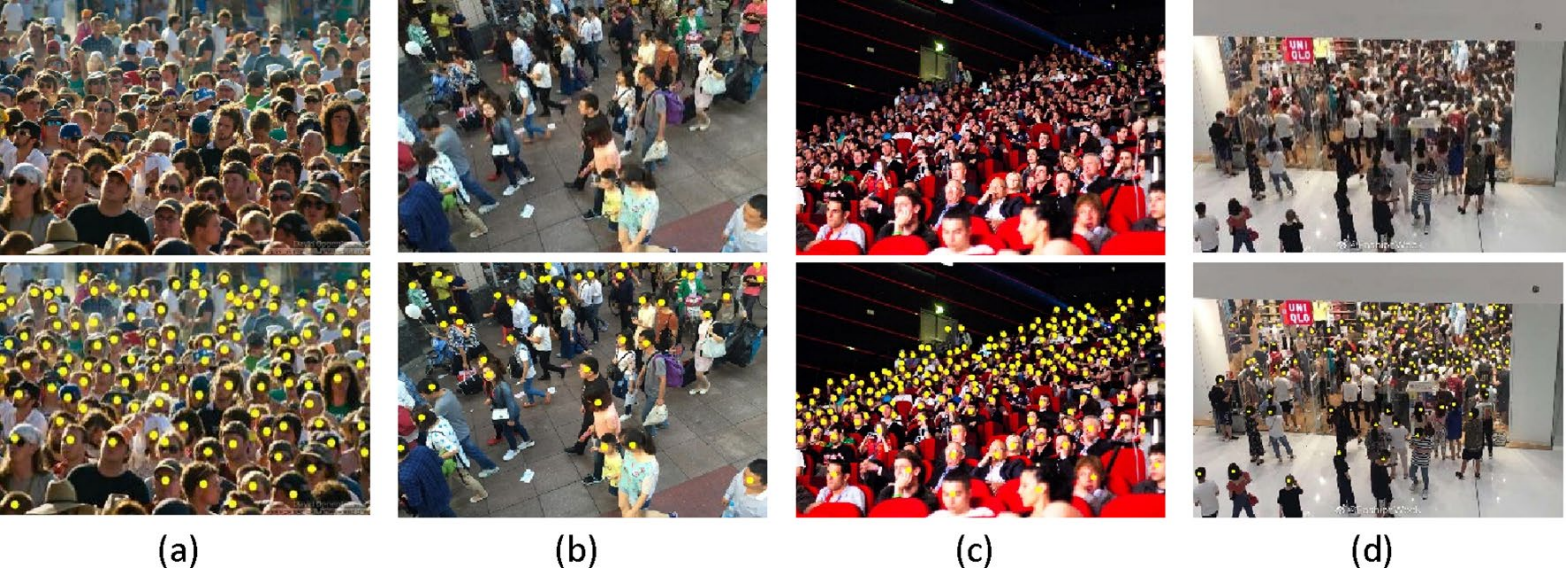
Visualization of crowd localization.

**Figure 5:**
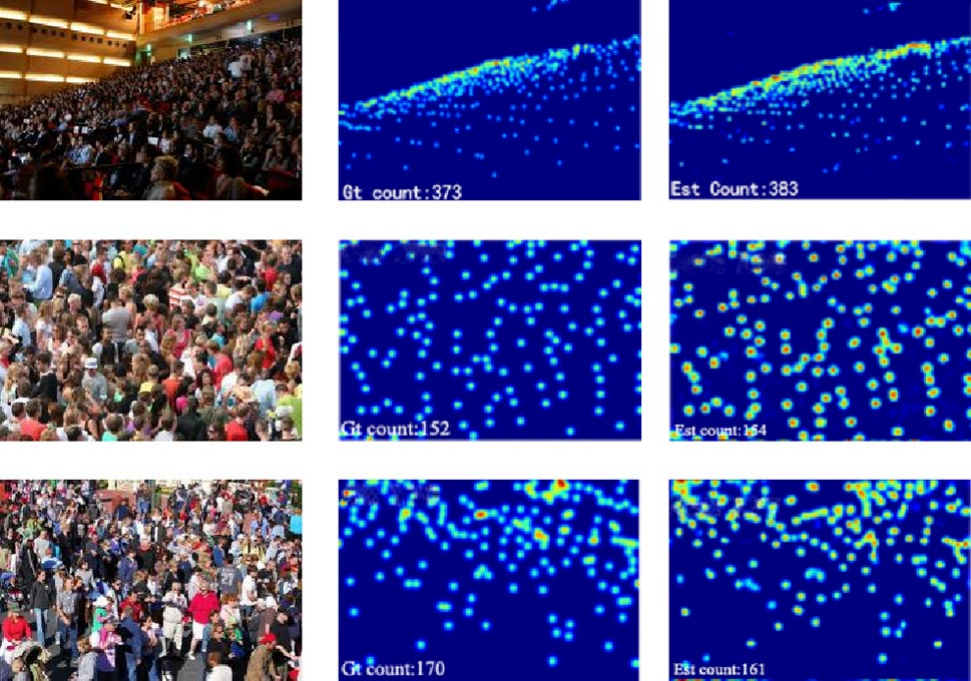
Visual comparisons of crowd counting on ShanghaiTech PartA. The first row is the sample images. The second row is the ground truth. The 3rd rows correspond to the estimated density maps from WSITrans.

Figure 4 shows two dense scenes and two sparse scenes, among them, (a) and (b) are from ShanghaiTech PartA and PartB, (c) is from UCF_QNRF, and (d) is from NWPU-Crowd. The visual comparisons of crowd counting on ShanghaiTech PartA are shown in Fig. 5.

## Conclusion

In this work, we propose a new architecture called WSITrans, which extracts features through an improved Transformer based on weakly supervised for an end-to-end trained crowd localization, while implementing crowd counting. A global maximum pooling operation is added at each stage of the Transformer backbone to extract and retain richer details of heads. We adopt weakly supervised learning to reduce complex pre-processing, and the position information is embedded into the aggregation features. It can greatly enhance the performance of WSITrans by the optimized adaptive threshold learner in the binarization module. In addition, extensive comparative experiments on three challenging datasets show that WSITrans is effective. In the future, we intend to use unsupervised learning to explore a lightweight crowd localization model and improve the efficiency of crowd analysis. In addition, the quality of the density map is further enhanced by using a generative adversarial network (GAN), so we will consider GAN for future research.

## Data Availability

The datasets generated during and/or analyzed during the current study are available from the corresponding author upon reasonable request.
